# Supplementary material for: Acoustic Wave‐Induced Stroboscopic Optical Mechanotyping of Adherent Cells
Source: Adv Sci (Weinh). 2024 Feb 28;11(16):2307929. doi: 10.1002/advs.202307929 (PMC11040383; doi:10.1002/advs.202307929)
Supplement: Supplementary file 1 — Supporting Information [file ADVS-11-2307929-s005.pdf]

ADVANCED SCIENCE
Open Access

# Supporting Information

for *Adv. Sci.*, DOI 10.1002/advs.202307929

Acoustic Wave-Induced Stroboscopic Optical Mechanotyping of Adherent Cells

*Thomas Combriat, Petter Angell Olsen, Silja Borring Låstad, Anders Malthe-Sørenssen, Stefan Krauss and Dag Kristian Dysthe**

# Supplementary Figures: Acoustic wave-induced stroboscopic optical mechanotyping of adherent cells

*Thomas Combriat Petter Angell Olsen Silja Borring Låstad Anders Malthe-Sørenssen Stefan Krauss Dag Kristian Dysthe*

Dr. Thomas Combriat
University of Oslo, Department of Physics, Njord Centre, P.O. Box 1048 Blindern, 0316 Oslo, Norway
University of Oslo, Hybrid Technology Hub, Institute of Basic Medical Sciences P.O. Box 1110 Blindern, 0317 OSLO, Norway
University of Oslo, Center for Computing in Science Education, P.O. Box 1048 Blindern, 0316 Oslo, Norway
Dr. Petter Angell Olsen
University of Oslo, Hybrid Technology Hub, Institute of Basic Medical Sciences P.O. Box 1110 Blindern, 0317 OSLO, Norway
Oslo University Hospital, Department of Immunology and Transfusion Medicine, P.O. Box 4950, Nydalen, 0424 Oslo, Norway
Silja Borring Låstad
University of Oslo, Department of Physics, Njord Centre, P.O. Box 1048 Blindern, 0316 Oslo, Norway
Pr. Anders Malthe-Sørenssen
University of Oslo, Department of Physics, Njord Centre, P.O. Box 1048 Blindern, 0316 Oslo, Norway
University of Oslo, Center for Computing in Science Education, P.O. Box 1048 Blindern, 0316 Oslo, Norway
Pr. Stefan Krauss
University of Oslo, Hybrid Technology Hub, Institute of Basic Medical Sciences P.O. Box 1110 Blindern, 0317 OSLO, Norway
Oslo University Hospital, Department of Immunology and Transfusion Medicine, P.O. Box 4950, Nydalen, 0424 Oslo, Norway
Pr. Dag Kristian Dysthe University of Oslo, Department of Physics, Njord Centre, P.O. Box 1048 Blindern, 0316 Oslo, Norway
Email adress: d.k.dysthe@fys.uio.no

## 1 Supplementary movies

The following videos are made available for demonstration purposes.

- co-culture_MDCK_MelJuSo: Slow-motion video of a co-culture of MDCK cells and MelJuSo cells oscillating at ultrasound frequency
- HeLa_sc: Slow-motion video of a culture of HeLa cells oscillating at ultrasound frequency
- HTC116_sc: Slow-motion video of a co-culture of HTC116 cells oscillating at ultrasound frequency
- MDCK_sc: Slow-motion video of a co-culture of MDCK cells oscillating at ultrasound frequency
- MelJuSo_sc: Slow-motion video of a co-culture of MelJuSo cells oscillating at ultrasound frequency
- `https://www.youtube.com/watch?v=4nQc6Gn7qws`: Overview video of the analysis process

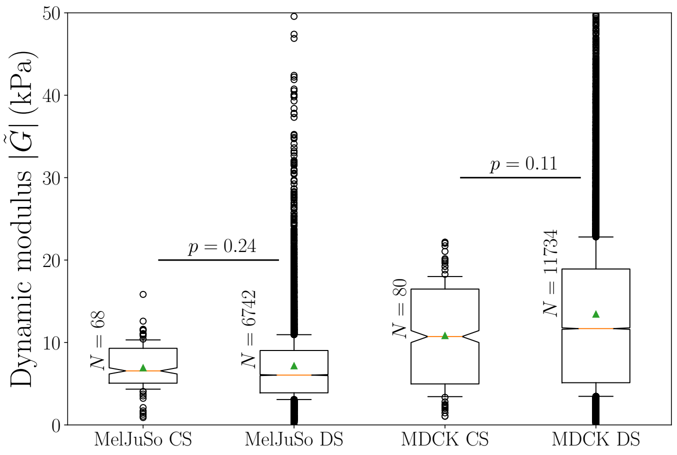


Figure S1: **Dynamic shear modulus $|\tilde{G}|$ boxplots calculated using cells (CS) and DIC-box (DS) as the statistical unit**. Data are extracted from one experiment done on a mixed population of cells (MelJuSo and MDCK). The number of statistical units, $N$, for each boxplot is a factor 100 larger for DIC-box than for cells. Despite this the two distributions for each cell type have very similar median and standard errors (denoted by the the notches) showing that DIC-boxes can be used as the statistical unit. The displayed $p$-values are the results of a Mann-Whitney U test.

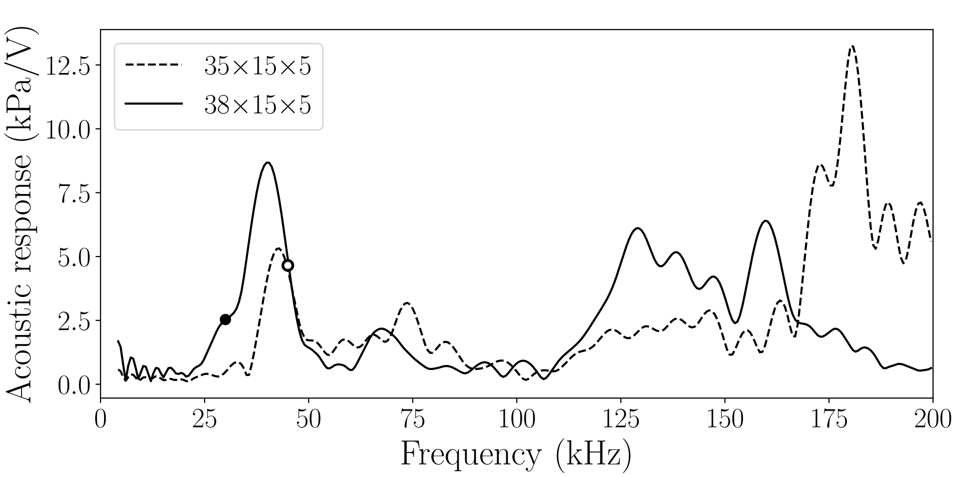


Figure S2: Calibration of the two transducers used in this study. The dots denote the frequencies used for each transducer.
